# Swift action increases the success of population reinforcement for a declining prairie grouse

**DOI:** 10.1002/ece3.3776

**Published:** 2018-01-15

**Authors:** Michael A. Hardy, Scott D. Hull, Benjamin Zuckerberg

**Affiliations:** ^1^ Department of Forest & Wildlife Ecology University of Wisconsin‐Madison Madison WI USA; ^2^ Office of Applied Science Wisconsin Department of Natural Resources Madison WI USA

**Keywords:** augmentation, population viability analysis, prairie‐chicken, restocking, translocation

## Abstract

Translocations have become an increasingly valuable tool for conservation in recent years, but assessing the successfulness of translocations and identifying factors that contribute to their success continue to challenge biologists. As a unique class of translocation, population reinforcements have received relatively little attention despite representing a substantial portion of translocation programs. Here, we conducted population viability analyses to quantify the effects of 216 reinforcement scenarios on the long‐term viability of four populations of Greater Prairie‐Chickens (*Tympanuchus cupido pinnatus*) in Wisconsin, USA, and used multiple linear regression to identify factors that had the greatest relative influence on population viability. We considered reinforcements from outside of the study area in addition to translocations among Wisconsin populations. We observed the largest decreases in site‐specific extinction probability and the largest increases in the number of sites persisting for 50 years when more vulnerable populations were targeted for reinforcement. Conversely, reinforcing the most stable sites caused the greatest reduction in regional extinction probability. We found that the number of translocated hens was a comparatively poor predictor of changes in long‐term population viability, whereas the earlier onset of reinforcement was consistently associated with the greatest increases in viability. Our results highlight the value of evaluating alternative reinforcement strategies a priori and considering the effects of reinforcement on metrics of long‐term population persistence.

## 1 INTRODUCTION

Conservation translocations, defined as “the intentional movement and release of a living organism where the primary objective is a conservation benefit (IUCN 2013),” are an important tool for restoring and enhancing populations of plants (Godefroid et al., 2011) and animals (Seddon, Griffiths, Soorae, & Armstrong, 2014). As conservation translocations have become increasingly common in recent years (Brichieri‐Colombi & Moehrenschlager, 2016), interest in identifying factors that contribute to their success and failure has given rise to the discipline of reintroduction biology (Seddon, Armstrong, & Maloney, 2007). The emergence of reintroduction biology has generated new recommendations for translocations such as increased collaboration between ecologists and managers (Sarrazin & Barbault, 1996), a priori identification of clearly articulated goals (Armstrong & Seddon, 2008; Seddon et al., 2007), and more rigorous approaches to planning and long‐term monitoring (Armstrong & Seddon, 2008; Fischer & Lindenmayer, 2000; Godefroid et al., 2011; Griffith, Scott, Carpenter, & Reed, 1989; Seddon, 1999). Despite efforts in developing comprehensive guidelines for planning and implementing translocations (IUCN 2013), there are still several unresolved issues on how to best evaluate a translocation program.

**Figure 1:**
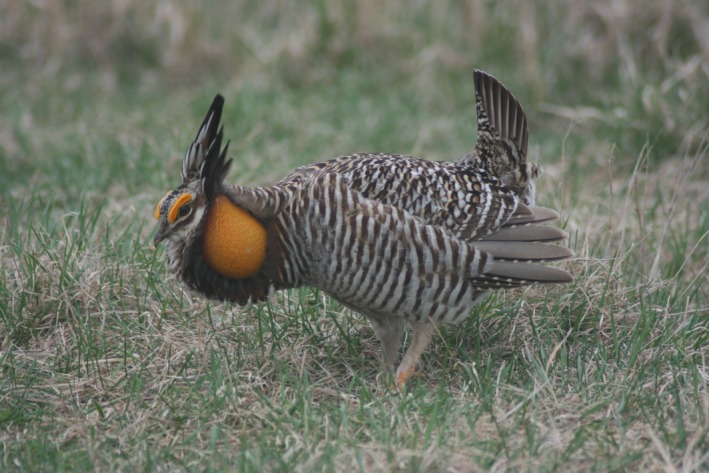
Greater Prairie‐Chicken (*Tympanuchus cupido pinnatus*) is a lek‐mating grouse species endemic to North American grasslands. Greater Prairie‐Chickens have exhibited substantial population declines throughout much of their range and are commonly the focus of intensive management efforts such as translocations. Photo © L. Kardash 2008

Regardless of which criteria are used to evaluate the success or failure of a translocation effort, any determination of success is only valid for the point in time when the population was assessed (Seddon, 1999), and a translocation initially deemed successful may ultimately “fail” in the future (Wolf, Griffith, Reed, & Temple, 1996). Thus, categorizing a translocation as successful in the short‐term may in fact be misleading, as it fails to consider metrics of long‐term persistence (Seddon, 1999). For example, translocations of Greater Prairie‐Chickens (*Tympanuchus cupido pinnatus*; Figure 1) to Illinois were associated with immediate increases in genetic diversity, fertility, and hatching success (Westemeier et al., 1998), but did not cause a substantial long‐term increase in population size despite the acquisition of additional grassland habitat (Bouzat et al., 2009). Similarly, translocations of prairie‐chickens to Wisconsin increased mtDNA diversity (Bateson et al., 2014), but populations have continued to decline following reinforcement. Consequently, both efforts could be considered successful to some extent, but the long‐term effects of translocations on the future viability of the recipient populations remain largely unknown. As the ultimate goal of many translocation programs (and indeed, much of conservation biology) is the long‐term persistence of viable populations (Seddon, 1999), there is a clear need to evaluate the potential effects of translocations over longer timescales and examine metrics such as estimated local and/or regional quasi‐extinction probability through formal population viability analysis (PVA; IUCN 2013).

Because translocations can be costly (Lindberg, 1992; Weise, Stratford, & van Vuuren, 2014), controversial (Hoegh‐Guldberg et al., 2008), and may have a comparatively small likelihood of success (Griffith et al., 1989), identifying factors that are likely to contribute to success is an essential component of designing an effective translocation program. Previous post hoc analyses have explored the effects of a wide variety of factors on translocation success, including habitat quality, number of animals released, number and duration of releases, and source population (Brichieri‐Colombi & Moehrenschlager, 2016; Fischer & Lindenmayer, 2000; Griffith et al., 1989; Wolf et al., 1996). However, there is no substitute for a priori assessment of potential translocation outcomes that consider the unique set of circumstances associated with a proposed translocation. Recent investigators have made important steps forward in this regard by employing model‐based approaches to optimize translocation strategies (Canessa, Hunter, McFadden, Marantelli, & McCarthy, 2014; Decesare et al., 2011; Rout, Hauser, & Possingham, 2007, 2009), yet the majority of translocation studies focus on short‐term population establishment and studies that explicitly compare multiple management strategies are still comparatively rare (Taylor et al., 2017). Although the value of statistically rigorous approaches to planning and evaluating translocations is well‐established (Armstrong & Reynolds, 2012; Armstrong & Seddon, 2008; Converse & Armstrong, 2016), the results from even the best‐designed translocation studies may be of limited use when planning or evaluating a different class of translocation.

There are four recognized classes of conservation translocation (IUCN 2013, Seddon et al., 2014), and the overwhelming majority of the translocation literature has either focused specifically on reintroductions (i.e., the release of organisms within their indigenous range where conspecifics are no longer present, IUCN 2013) or failed to distinguish between types of translocation (Brichieri‐Colombi & Moehrenschlager, 2016; Griffith et al., 1989; Wolf et al., 1996). This is hardly surprising, as reintroductions are by far the most common class of conservation translocation (Brichieri‐Colombi & Moehrenschlager, 2016; Fischer & Lindenmayer, 2000): in North America, reintroductions were the subject of nearly 70% of translocation studies published from 1955–2013 (Brichieri‐Colombi & Moehrenschlager, 2016). However, population reinforcement―the release of organisms into an existing population of conspecifics―is also quite common, representing nearly 27% of translocation projects during the same period (Brichieri‐Colombi & Moehrenschlager, 2016). Nonetheless, there are comparatively few syntheses that specifically address reinforcements (Champagnon, Elmberg, Guillemain, Gauthier‐Clerc, & Lebreton, 2012).

While much of the literature on reintroductions might be relevant to reinforcements, releasing organisms into an existing population is fundamentally different than establishing a “new” population and the factors that promote successful establishment are not necessarily the same as those that enhance population persistence or spread (Bright & Smithson, 2001). For example, managers planning a reintroduction must decide where the population should be established (i.e., core, periphery, or outside the species’ historic range), which can have a substantial effect on the likelihood of success (Wolf et al., 1996), and there is often considerable uncertainty regarding the suitability of reintroduction sites prior to releases (Armstrong & Wittmer, 2011; McCarthy, Armstrong, & Runge, 2012). Conversely, reinforcements are concerned with enhancing the persistence of an existing population, and as a result, do not have the same barriers of establishment (Armstrong & Seddon, 2008). Furthermore, managers often have a better understanding of the factors thought to limit populations that are targeted for reinforcement, facilitating more effective mitigation of threats prior to translocations. Finally, other aspects of population reinforcements that may not be as relevant to reintroductions (e.g., timing of conservation measures; Martin et al., 2012; Martin, Camaclang, Possingham, Maguire, & Chadès, 2016) might play a key role in avoiding local extinction.

As population reinforcement is one of the most common tools proposed and implemented for declining prairie grouse, we used a count‐based PVA framework to facilitate an a priori evaluation of 216 alternative reinforcement scenarios for four Wisconsin populations of Greater Prairie‐Chickens that vary substantially in their extinction risk. We considered two factors that have been previously associated with translocation success (number of individuals released and number of releases) and one potentially important factor that has received comparatively little attention in reintroduction biology―timing of reinforcements―and identified which factors were most strongly associated with long‐term population persistence. We considered three alternative metrics of long‐term population viability to demonstrate that optimum reinforcement strategies can differ depending on the definition of success, underscoring the importance of clearly identifying goals for reinforcement projects. Our approach uses long‐term data to address many of the challenges associated with evaluating reinforcements and is flexible enough to be applied during the planning stage of translocations for a wide variety of taxa. Moreover, a simulation‐based approach such as the one presented here could facilitate adaptive management by enabling managers to compare post‐release population performance to model expectations and modify release strategies as necessary to optimize future reinforcement efforts.

## 2 MATERIALS AND METHODS

### 2.1 Study system

**Figure 2:**
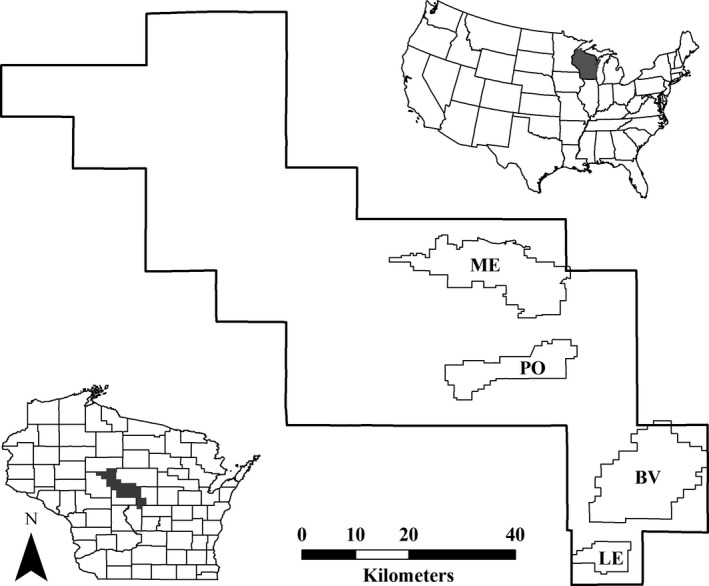
Location of four focal sites (Buena Vista, BV; Paul Olson, PO; Leola, LE; Mead, ME) in the Central Wisconsin Grassland Conservation Area, Wisconsin, USA

In Wisconsin, USA, Greater Prairie‐Chickens have declined from an estimated ≈54,000 birds in 1930 to <1,000 individuals today. Coincident with this long‐term population decline, prairie‐chickens have experienced a significant range contraction due to extensive conversion of grassland habitat to other land uses and have concurrently lost substantial genetic diversity since the early 1950s (Bellinger, Johnson, Toepfer, & Dunn, 2003; Johnson & Dunn, 2006). Today, prairie‐chickens are state‐listed as threatened in Wisconsin and are largely restricted to four state‐managed properties in the Central Wisconsin Grassland Conservation Area (CWGCA): Buena Vista Marsh Wildlife Area (BV), Paul J. Olson Wildlife Area (PO), Leola Marsh Wildlife Area (LE), and George W. Mead Wildlife Area (ME; Figure 2). Although these core properties are actively managed for grassland‐dependent species via techniques such as mowing, grazing, and prescribed burning, much of the remainder of the CWGCA still remains in agricultural production. Moreover, agricultural land use in the CWGCA has increasingly shifted from pasture and other grassy habitats to irrigated row crops in recent decades (Anderson & Toepfer, 1999). Consequently, prairie‐chickens have been extirpated from the northern portion of the study area since the mid‐1990s and the remaining populations have become progressively more isolated. Concerns about loss of genetic diversity prompted managers to reinforce the Buena Vista population with a total of 110 hens from Minnesota between 2006–2009. Although the effects of previous reinforcements on long‐term population viability remain unclear, additional reinforcement efforts remain a viable, if not preferred, management option.

### 2.2 Baseline viability analysis

As a first step in assessing long‐term viability of prairie‐chickens in the CWGCA, we treated spring counts of dancing males as an index of population size and conducted a count‐based PVA incorporating annual stochasticity in growth rates (Morris & Doak, 2002) for each of the four focal sites (Figure 2). Although count data are often readily available to managers, count‐based PVA methods require long‐term datasets to obtain reliable estimates of variation in population growth, and therefore are not often used to model translocations. In our case, count data were available from 1950–2015 for Buena Vista and Leola, 1962–2015 for Paul Olson, and 1969–2015 for Mead (Table S1). Because surveys were not conducted at Mead during 1984 and 1986, we estimated male counts during those two years as the midpoint between counts from the preceding and following year. Prairie‐chickens have evolved a highly competitive polygynous mating strategy wherein the majority of males will not reproduce in any given year. In contrast, most, if not all, females typically have the opportunity to breed with dominant males; we therefore assumed a 1:1 sex ratio (Bellinger et al., 2003; McNew, Gregory, Wisely, & Sandercock, 2012; Wisdom & Mills, 1997) and conducted all analyses for breeding females. We first calculated annual site‐specific population growth rates (i.e., log(λ_*t*_)) for each of the four focal sites. Following Morris and Doak (2002), we fit two density‐dependent models (theta‐logistic, Ricker‐logistic) and a density‐independent model to the observed growth rates and selected the best‐fitting model for each site based on Akaike's information criterion corrected for small sample size (AIC_*c*_; Burnham & Anderson, 2002).

We then used the parameter estimates for each site to simulate the fates of 10,000 populations for 50 years into the future and calculated three metrics of long‐term viability that are of interest to managers in the CWGCA: cumulative probability of quasi‐extinction at each site, cumulative probability of regional quasi‐extinction (i.e., all four sites falling below the quasi‐extinction threshold), and, in cases where ≥1 site remained extant, the average number of sites persisting in the CWGCA (i.e., 1–4). We defined the quasi‐extinction threshold as the minimum number of breeding females below which the population is likely to be critically and immediately imperiled (Ginzberg, Slobodkin, Johnson, & Bindman, 1982), and used a quasi‐extinction threshold of *N*_*x*_ = 20 hens for all analyses (Morris & Doak, 2002). For Buena Vista and Paul Olson, we used the 2015 counts (133 and 90 hens, respectively) as the initial population size for the simulations. Leola and Mead were already below the quasi‐extinction threshold in 2015 (17 and 13 hens, respectively), and will likely require immediate intervention to persist; we therefore added 20 hens to each site prior to conducting simulations to represent such an intervention. We treated each site as independent of the others and did not model movement of hens because (1) radiotelemetry data indicate that there is little to no movement among the core sites (Wisconsin Department of Natural Resources, *unpublished data*) and (2) any contributions of immigration and emigration to population growth are implicitly included in the site‐specific log(λ_*t*_) values. Finally, we quantified the uncertainty associated with parameter estimates using a nonparametric bootstrap approach. Briefly, we resampled with replacement the observed log(λ_*t*_) values for each site, re‐fit the model, and used the new parameter estimates to simulate an additional 10,000 populations. We repeated this process 10,000 times to obtain quantiles and 95% confidence intervals.

### 2.3 Reinforcement scenarios

After establishing our baseline estimate of viability, we conducted additional PVAs under each of 216 alternative reinforcement scenarios split into two sets based on the source of translocated hens. Although prairie‐chickens have declined throughout much of their range, stable or increasing populations that could potentially act as sources for translocations do exist in several other states (e.g., Minnesota; Johnson, Schroeder, & Robb, 2011) and hens have been translocated from Minnesota to Wisconsin in the past. For the first set of scenarios, we simulated translocating hens from one of these outside sources and adding them to one of the CWGCA populations prior to breeding during ≥1 time step, and did not model the effects of removal on the donor population. We varied the number of releases (a single reinforcement event vs. decadal reinforcements) and level of effort (20 vs. 100 hens per reinforcement) among scenarios. Because timely efforts can be crucial for effective conservation (Martin et al., 2012), we also investigated the effects of delayed action by varying the onset of translocation efforts (5, 10, 15, 20, 25, 30, 35, 40, or 45 years into the future), resulting in 36 scenarios per site (144 scenarios total).

For the second set of scenarios, we assumed that no outside source of hens was available and instead simulated translocating hens from one of the two largest CWGCA populations (Buena Vista or Paul Olson) to one of the smaller populations (Leola or Mead) while considering effects on both the donor and recipient populations. Translocations only occurred if the donor population had ≥100 hens prior to breeding in that year and the recipient population was still above the quasi‐extinction threshold (i.e., no “rescue effects”). As above, we varied the frequency and onset of translocations. However, we always simulated moving 20 rather than 100 hens because of the comparatively small size of the donor populations. Consequently, analyses of site‐specific extinction probability did not consider level of effort, but instead considered donor (Buena Vista or Paul Olson) or recipient population (Leola or Mead), resulting in an additional 72 scenarios.

Radiotelemetry data collected in our study area from 2007–2009 indicate that only 10 of 110 translocated hens either dispersed out of detection range or had failed transmitters, only four translocated hens died within 7 days of release, breeding season survival estimates were similar among native and released hens in 2 of 3 years, and survival was similar during both overwinter periods (Wisconsin Department of Natural Resources, *unpublished data*). Consequently, our reinforcement scenarios assumed that all translocated hens remained in the population where they were released and that translocated hens had identical survival to native hens. However, we recognize that appreciable numbers of translocated prairie‐chickens may permanently emigrate from their release location (Kemink & Kesler, 2013) and that translocated individuals may have substantially lower survival (Carrlson, Kesler, & Thompson, 2014). We therefore note that in practice, larger release cohorts may be necessary to account for differential survival and permanent emigration of released hens.

### 2.4 Statistical analysis

To quantify the relative benefits of different reinforcement scenarios, we first calculated three metrics of success (i.e., change in site‐specific extinction probability, regional extinction probability, and number of extant sites in the CWGCA at year 50) for each scenario relative to the baseline scenario. Specifically, we subtracted the baseline estimate from the alternative estimate for each of the 10,000 bootstrap replicates, yielding a vector of changes for each scenario‐metric combination. We then calculated the mean value of each vector, with the expectation that the mean would be negative if the scenario decreased extinction probability or number of extant sites, positive if the scenario increased extinction probability or number of extant sites, and zero if the reinforcement had no long‐term effect. We were interested not only in identifying the most effective reinforcement scenario, but also in identifying factors that were consistently associated with increased long‐term viability among multiple scenarios. We therefore used multiple linear regression to assess the relative importance of effort, frequency, onset, recipient population, and donor population on the mean change to each response metric associated with each reinforcement scenario. To avoid the confounding effects of effort, donor, and recipient populations, we analyzed translocations from an outside source and translocations from within Wisconsin separately. To identify factors that were consistently strong predictors of change in population viability, we first fit a set of eight or 16 candidate models for site‐specific and regional metrics, respectively, and ranked models based on AIC_*c*_. We then quantified the relative influence of each factor on changes to population viability by calculating partial *R* ^2^ values (i.e., the proportion of residual variation explained) for each predictor in each of the candidate models. We present only the best‐supported models here; all candidate models and their associated rankings are presented as supporting information. All analyses were conducted in R version 3.3.1 (R Core Team 2016).

## 3 RESULTS

### 3.1 Baseline viability analysis

**Figure 3:**
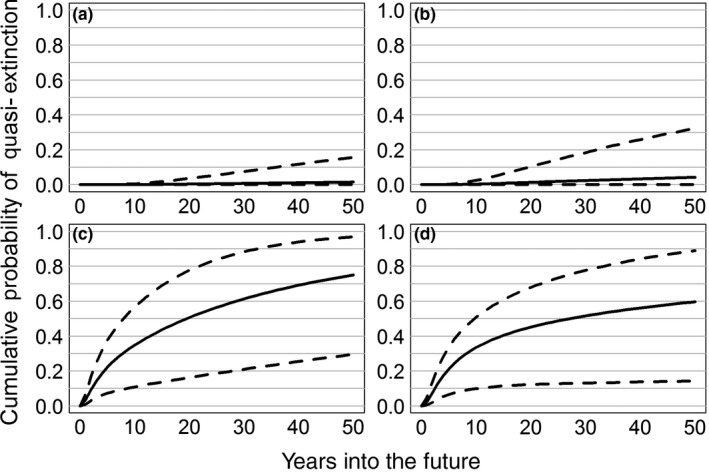
Cumulative probability of quasi‐extinction for four populations of Greater Prairie‐Chickens (Buena Vista, a; Paul Olson, b; Leola, c; Mead, d) in the Central Wisconsin Grassland Conservation Area, Wisconsin, USA. Solid lines denote average extinction probability and dashed lines denote 95% confidence intervals calculated from 10,000 nonparametric bootstrap replicates

**Figure 4:**
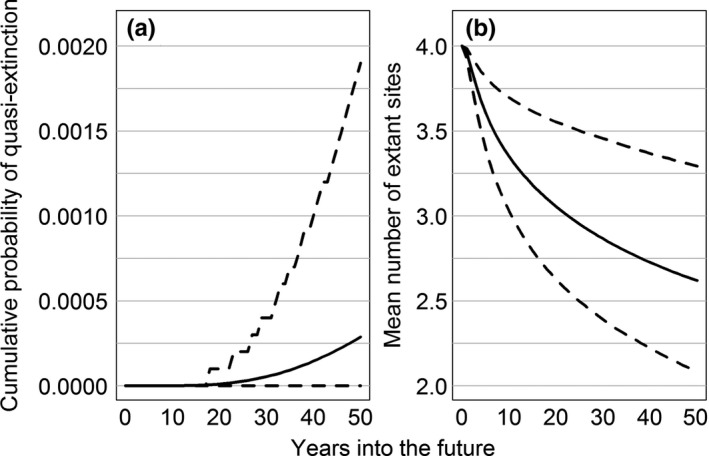
Cumulative probability of quasi‐extinction for Greater Prairie‐Chickens in the Central Wisconsin Grassland Conservation Area, Wisconsin, USA. (a) and the mean number of extant breeding sites with ≥20 hens (b). Solid lines denote average values and dashed lines denote 95% confidence intervals calculated from 10,000 non‐parametric bootstrap replicates

We found that the best‐supported model for each site was the Ricker‐logistic model (Ricker, 1954):

$$\text{log}\left(\lambda_{t}\right)=r\left(1-\frac{N_{t}}{K}\right)+\;\varepsilon_{t}$$

where *r *= the intrinsic population growth rate, *N*_*t*_ = population size at time *t*,* K *= carrying capacity, and ε_*t*_ accounts for annual variation in population growth. ε_*t*_ is normally distributed with mean = 0 and variance = σ^2^, with σ^2^ representing the amount of environmental stochasticity. Estimated probability of quasi‐extinction in the next 50 years varied substantially among the four sites (Figure 3). Buena Vista was the most likely to persist for 50 years (mean quasi‐extinction probability = 0.015, 95% CI = 0–0.157), followed by Paul Olson (mean = 0.042, 95% CI = 0–0.323). In contrast, both Leola (mean = 0.749, 95% CI = 0.295–0.968) and Mead (mean = 0.596, 95% CI = 0.142–0.888) were highly vulnerable and very likely to become extirpated within the next 50 years, even with the immediate addition of 20 hens to each site. Not surprisingly, estimates of intrinsic population growth rates (*r*) were greatest at Buena Vista, followed by Paul Olson, Leola, and Mead. However, variability in the long‐term growth rate (σ/*r*) followed the opposite trend. Buena Vista exhibited the least variation in population growth, whereas variability was highest at Mead (Figure S1). Taking into account population size, average growth rate, and variability in annual growth, the four sites fall along a continuum of increasing vulnerability with Buena Vista being the least vulnerable, followed by Paul Olson, Leola, and Mead. At the regional scale, it is highly unlikely that all four sites will simultaneously fall below the quasi‐extinction threshold within 50 years (mean < 0.001, 95% CI = 0–0.002; Figure 4a). Nonetheless, it is also highly unlikely that all four sites will persist for the next 50 years (mean number of extant sites = 2.619, 95% CI = 2.083–3.294; Figure 4b).

### 3.2 Translocations from an outside source

**Figure 5:**
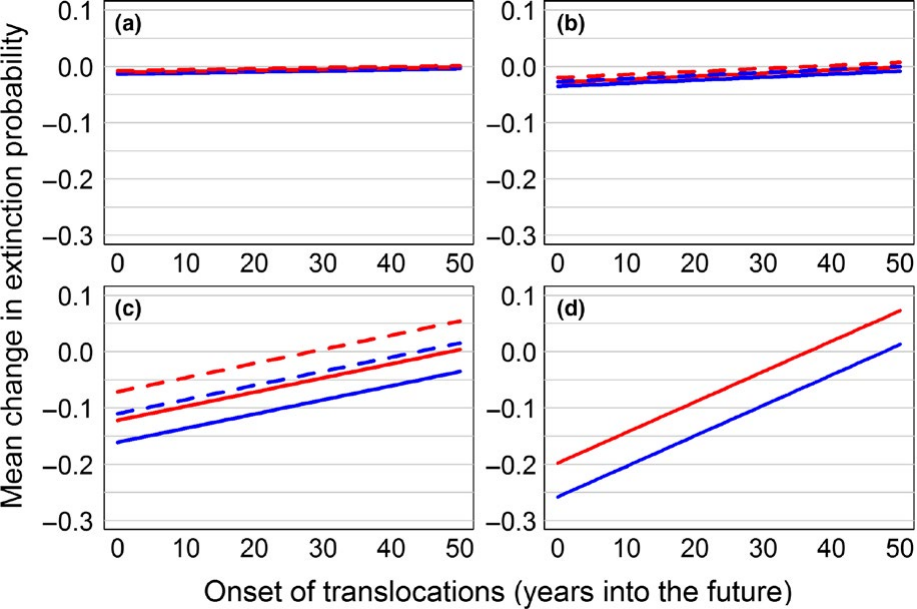
Simulated effects of translocations of Greater Prairie‐Chickens originating from an outside source on site‐specific quasi‐extinction probability for four populations (Buena Vista, a; Paul Olson, b; Leola, c; Mead, d) in the Central Wisconsin Grassland Conservation Area, Wisconsin, USA. Solid lines denote decadal translocations, dashed lines denote a single translocation, blue denotes 100 translocated hens, and red denotes 20 translocated hens

Translocations from an outside source led to the greatest reduction in extinction probability at the most vulnerable sites (Leola and Mead; Figure 5). The top models for Buena Vista (∆AIC_*c*_ ≥ 7.72, *w*_*i *_= 0.97, *R* ^2^_adj _= .63), Paul Olson (∆AIC_*c*_ ≥ 9.09, *w*_*i*_ **= 0.99, *R* ^2^_adj _= .70), and Leola (∆AIC_*c*_ ≥ 6.35, *w*_*i*_ **= 0.96, *R* ^2^_adj_ = .59) all included level of effort (i.e., number of translocated hens), frequency of reinforcement, and onset of reinforcement, whereas the top model for Mead (∆AIC_*c*_ ≥ 0.76, *w*_*i*_ **= 0.59, *R* ^2^_adj_ = .70) included only level of effort and onset (Table S2). In all cases, the benefits of reinforcement increased with more translocated hens, earlier action, and/or decadal reinforcements rather than a single reinforcement; all of these relationships were stronger at more vulnerable sites (Table 1). In terms of relative variable importance, level of effort accounted for the least amount of residual variation in extinction probability at each of the four focal sites (partial *R* ^2 ^= .22–.28), whereas onset of population reinforcements consistently explained the most variation (partial *R* ^2 ^= .46–.69; Table 2).

**Table 1:** Coefficient estimates (*SE*) for predictors of the effects of population reinforcement on three metrics of long‐term viability of Greater Prairie‐Chickens in the Central Wisconsin Grassland Conservation Area, Wisconsin, USA

Variable	Site‐specific extinction probability^a^				Regional viability^b^	
	BV	PO	LE	ME	Extinction probability	# populations
Translocations from an outside source						
Intercept	−0.0032 (0.0007)	−0.0065 (0.0017)	−0.0086 (0.0111)	−0.0625 (0.0121)	−7.012e‐05 (1.120e‐05)	−0.0176 (0.0099)
Effort (100 hens)^c^	−0.0027 (0.0008)	−0.0071 (0.0020)	−0.0390 (0.0129)	−0.0601 (0.0171)	−4.469e‐05 (9.146e‐06)	0.0303 (0.0089)
Frequency (decadal)^d^	−0.0030 (0.0008)	−0.0083 (0.0020)	−0.0508 (0.0129)	‐	−2.068e‐05 (9.146e‐06)	‐
Onset of translo‐cations	0.0026 (0.0004)	0.0074 (0.0010)	0.0344 (0.0065)	0.0741 (0.0087)	4.137e‐05 (4.589e‐06)	−0.0399 (0.0045)
Recipient population^e^						
PO	‐	‐	‐	‐	1.108e‐06 (1.293e‐05)	0.0209 (0.0126)
LE	‐	‐	‐	‐	8.312e‐05 (1.293e‐05)	0.0601 (0.0126)
ME	‐	‐	‐	‐	6.562e‐05 (1.293e‐05)	0.0939 (0.0126)
Translocations from within Wisconsin						
Intercept	0.0022 (0.0001)	0.0037 (0.0002)	−0.0105 (0.0040)	0.0037 (0.0097)	9.618e‐05 (3.656e‐06)	−0.0772 (0.0055)
Frequency (decadal)^d^	‐	0.0007 (0.0003)	−0.0291 (0.0046)	−0.0348 (0.0112)	‐	0.0356 (0.0055)
Onset of translo‐cations	−0.0005 (0.0001)	−0.0008 (0.0001)	0.0135 (0.0023)	0.0426 (0.0057)	‐	−0.0249 (0.0028)
Recipient population (ME)^f^	‐	‐	‐	‐	‐	0.0249 (0.0055)
Donor population (PO)^e^	‐	‐	0.0099 (0.0046)	0.0260 (0.0112)	‐	−0.0135 (0.0055)

a Study sites: Buena Vista (BV), Paul Olson (PO), Leola (LE), Mead (ME).

b Probability of all four study populations going extinct and average number of extant populations 50 years into the future.

c Reference level: 20 hens.

d Reference frequency: single.

e Reference population: Buena Vista.

f Reference population: Leola.

**Table 2:** Partial *R* ^2^ values for predictors of the effects of population reinforcement on three metrics of long‐term viability of Greater Prairie‐Chickens in the Central Wisconsin Grassland Conservation Area, Wisconsin, USA

Variable	Site‐specific extinction probability^a^				Regional viability^b^	
	BV	PO	LE	ME	Extinction probability	# populations
Translocations from an outside source						
Level of effort	0.25	0.28	0.22	0.27	0.15	0.08
Frequency of translocations	0.30	0.35	0.33	‐	0.04	‐
Onset of translocations	0.55	0.62	0.46	0.69	0.37	0.37
Recipient population^c^	‐	‐	‐	‐	0.33	0.32
Translocations from within Wisconsin						
Frequency of translocations	‐	0.15	0.56	0.23	‐	0.38
Onset of translocations	0.42	0.47	0.51	0.64	‐	0.55
Recipient population^d^	‐	‐	‐	‐	‐	0.23
Donor population^e^	‐	‐	0.13	0.14	‐	0.08

a Study sites: Buena Vista (BV), Paul Olson (PO), Leola (LE), Mead (ME).

b Probability of all four study populations going extinct and average number of extant populations 50 years into the future.

c Possible recipient population: BV, PO, LE, or ME.

d Possible recipient population: LE or ME.

e Possible donor population: BV or PO.

The best‐supported model for regional extinction probability (∆AIC_*c*_ ≥ 3.03, *w*_*i*_ **= 0.82, *R* ^2^_adj _= .54, Table S3) included level of effort, frequency and onset of reinforcements, and recipient population. More translocated hens, decadal reinforcements, and earlier onset of translocations were associated with reduced extinction probability. Moreover, the greatest benefits occurred when hens were translocated to Buena Vista, with reinforcements becoming increasingly less effective at more vulnerable sites (Figure S2, Table 1). Onset of translocations was the best predictor of change to extinction probability (partial *R* ^2 ^= .37), although recipient population was nearly as important (partial *R* ^2 ^= .33). Conversely, level of effort and frequency of reinforcements accounted for comparatively little residual variation (partial *R* ^2 ^= .15 and .04, respectively; Table 2). The best‐supported model for number of extant populations included level of effort, onset of translocations, and recipient population (∆AIC_*c*_ ≥ 1.45, *w*_*i *_= 0.67, *R* ^2^_adj _= .52, Table S4); more translocated hens, earlier onset, and bolstering the most vulnerable sites increased the number of extant populations (Figure S3, Table 1). Similar to regional extinction probability, early onset of reinforcement efforts was most important for increasing the number of extant populations remaining in the CWGCA after 50 years (partial *R* ^2 ^= .37), recipient population was nearly as important (partial *R* ^2 ^= .32), and level of effort was a relatively poor predictor of changes to long‐term viability (partial *R* ^2 ^= .08; Table 2).

### 3.3 Translocations from within the study region

**Figure 6:**
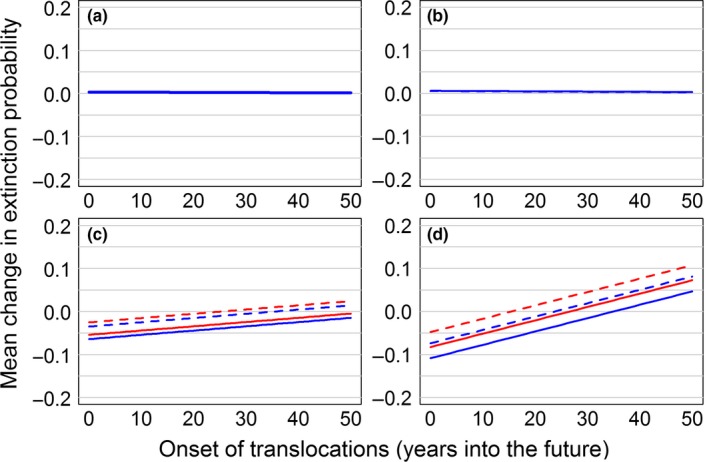
Simulated effects of translocations of Greater Prairie‐Chickens from within the study area on site‐specific quasi‐extinction probability for four populations (Buena Vista, a; Paul Olson, b; Leola, c; Mead, d) in the Central Wisconsin Grassland Conservation Area, Wisconsin, USA. Solid lines denote decadal translocations, dashed lines denote a single translocation; in panels c, d, blue denotes translocations from Buena Vista and red denotes translocations from Paul Olson

The overall patterns we observed when considering translocations among Wisconsin populations were generally similar, but with different effects on donor versus recipient populations (Figure 6, Table S5). For Buena Vista, the top model suggested that only onset of translocation effort had a substantial influence on extinction probability (∆AIC_*c*_ ≥ 1.74, *w*_*i *_= 0.52, *R* ^2^_adj _= .40), with earlier onset of translocations from Buena Vista having a slight detrimental effect regardless of translocation frequency or recipient population (Table 1). The top model for Paul Olson (∆AIC_*c*_ ≥ 1.17, *w*_*i *_= 0.53, *R* ^2^_adj _= .48) included frequency as well as onset; earlier and more frequent translocations both had a negative effect on persistence at Paul Olson (Table 1). Although onset was the most important predictor of change in extinction probability at both Buena Vista and Paul Olson (partial *R* ^2 ^= .42 and .47, respectively; Table 2), the increase to extinction probability was comparatively small in all cases (mean = 9.6 × 10^−5^, *SD *= 3.1 × 10^−5^). The best‐supported models at both Leola (∆AIC_*c*_ ≥ 2.22, *w*_*i *_= 0.75, *R* ^2^_adj _= .68) and Mead (∆AIC_*c*_ ≥ 2.88, *w*_*i *_= 0.78, *R* ^2^_adj _= .66) included donor population, reinforcement frequency, and onset of reinforcements (Table 1). As expected, increased frequency and earlier onset of reinforcements decreased extinction probability at both sites (Table 1). Additionally, translocations from Buena Vista had a greater benefit compared to translocations from Paul Olson, presumably because Buena Vista (as the most stable site) was more likely than Paul Olson to persist at or above the requisite threshold of 100 hens that would allow a translocation of hens to a more vulnerable site. However, it should be noted that donor population was not a particularly strong predictor at either site (partial *R* ^2 ^= .13 and 0.14 for Leola and Mead, respectively; Table 2). Although onset was by far the best predictor of change at Mead (partial *R* ^2 ^= .64), frequency of translocations accounted for slightly more residual variance than onset at Leola (partial *R* ^2 ^= .56 vs. .51; Table 2).

Of the 16 candidate models for regional quasi‐extinction probability, the null model received the most support (∆AIC_*c*_ ≥ 1.45, *w*_*i *_= 0.67, Table S6). In short, any effort involving the removal of hens from Buena Vista or Paul Olson was likely to have a negative effect on long‐term regional persistence. Moreover, translocating hens from Buena Vista or Paul Olson to Leola or Mead was also quite likely to have a negative effect on the average number of extant populations in the CWGCA (Figure S4). Although all of the scenarios reduced the average number of extant populations to some degree, the best‐supported model (∆AIC_*c*_ ≥ 3.76, *w*_*i *_= 0.87, *R* ^2^_adj _= .67) suggested that frequency and onset of translocations, donor, and recipient populations all had a substantial influence on change (Table 1, Table S7). Onset of translocations accounted for the greatest amount of residual variation (partial *R* ^2 ^= .55), followed by frequency of translocations (partial *R* ^2 ^= .38), recipient population (partial *R* ^2 ^= .23), and donor population (partial *R* ^2 ^= .08).

## 4 DISCUSSION

Translocations have become an increasingly important conservation tool in recent decades. Faced with numerous trade‐offs, scarce resources for conservation, and limited windows for effective action, there is a pressing need for a more robust evaluation of future translocation efforts (Armstrong & Seddon, 2008). Although there is a substantial body of literature on conservation translocations, intrinsic differences between reintroduction and reinforcement and inconsistent criteria for evaluation make generalizations about translocation success difficult, if not impossible. In particular, the results of post hoc analyses based on monitoring data collected over short time periods may be misleading, and the usefulness of such results to inform future translocation efforts may be limited, particularly if applied to a different class of translocation such as population reinforcement.

While early comparative analyses of translocation success suggest that translocations tend to be more successful when more individuals are released (Fischer & Lindenmayer, 2000; Griffith et al., 1989; Wolf et al., 1996), other studies of both reintroductions (Taylor, Jamieson, & Armstrong, 2005) and reinforcements (Van Houtan, Halley, van Aarde, & Pimm, 2009) have reported success with small release cohorts, and the perceived positive relationship between founder size and translocation success may in fact be confounded by other factors (Armstrong & Wittmer, 2011). Small populations are subjected to a suite of threats that make them relatively more vulnerable to extinction compared to larger populations (Caughley, 1994), and ensuring successful establishment of founders (e.g., reintroduced populations) falls chiefly within the realm of Caughley's “small‐population paradigm” (Armstrong & Seddon, 2008). Conversely, reinforcements are primarily concerned with promoting the persistence or expansion of populations that have already survived the establishment phase and are most likely regulated primarily by other factors (e.g., habitat quality or density dependence), and therefore, in theory, align more closely with the “declining population paradigm” (i.e., diagnosing and halting a population decline; Caughley, 1994). In practice, however, populations that are the targets of reinforcement efforts are often small as well as declining, and thus release cohort size is likely relevant for both reinforcements and reintroductions. In either case, the importance of release cohort size could reflect the degree to which the population in question is susceptible to small‐population effects (e.g., Allee effects, demographic stochasticity).

Although we found that the simulated release of 100 hens often led to a greater reduction in extinction probability than 20 hens, particularly at more vulnerable sites, the number of translocated individuals was generally a poor predictor of reinforcement success for all three metrics of population persistence, and in all but one case was the least important factor (Table 2). In our simulations, the addition of 100 hens consistently caused populations to greatly exceed carrying capacity (*K*) immediately following a reinforcement event, followed by a swift decline back to *K*; this pattern was most distinct at less vulnerable sites (i.e., Buena Vista and Paul Olson), but far less pronounced with additions of only 20 hens. We therefore conclude that the assumption that translocating more individuals will lead to a more desirable outcome may not always hold true, and reinforcing existing populations with large numbers of individuals without a concurrent increase in *K* (e.g., through extensive habitat management) may not be an efficient use of conservation resources unless the recipient populations are likely to become quickly extirpated without intervention.

In contrast to the number of released hens, the onset of reinforcement efforts was consistently among the most important predictors of translocation success. Indeed, onset explained the greatest amount of variation in long‐term viability in all but one case (i.e., site‐specific extinction probability at Mead, with translocations from Buena Vista or Paul Olson; Table 2). While the number of releases has been previously identified as a potentially important predictor of translocation success (Wolf et al., 1996), the timing of translocations has received comparatively little attention in reintroduction biology. However, the potentially tragic consequences of delayed conservation measures have been documented elsewhere (Lindenmayer, Piggott, & Wintle, 2013; Martin et al., 2012, 2016), and our results suggest that prompt action can substantially increase long‐term reinforcement success, particularly at vulnerable sites. We suspect that early onset of translocations will be of greater importance to population reinforcement than to reintroduction, but recent research suggests that timing can also be an important factor to consider in managed relocation programs (McDonald‐Madden, Runge, Possingham, & Martin, 2011).

Although the timing of reinforcement efforts was clearly important, the choice of a recipient population was also a key consideration. In terms of site‐specific persistence, the benefits of any given reinforcement strategy were strongest at the more vulnerable sites; whereas Buena Vista and Paul Olson did not benefit appreciably from reinforcement, Leola and Mead experienced substantial decreases in mean extinction probability (Figure 5). Similarly, bolstering the most vulnerable sites led to the greatest increases in the number of populations remaining in the CWGCA after 50 years (Figure S3). Conversely, buffering Buena Vista or Paul Olson provided the greatest boost to regional persistence (Figure S2) and recipient population explained nearly as much variation in regional extinction probability as onset of translocations (Table 2). While the absolute decrease in regional extinction probability was small in all cases, we note that even the baseline probability of regional extinction was quite low (<0.001) in the CWGCA and the benefits of reinforcing the “best” sites might be more significant in systems that are more vulnerable to regional population collapse. These results further highlight the importance of clearly defined goals for translocation programs, as the most effective translocation strategy may differ depending on how managers define “success.”

Finally, the choice of a donor population and the effects of removing individuals for translocation must be considered carefully, particularly if available donor populations are comparatively small (e.g., Bustamante, 1996, 1998). In our second set of scenarios, we simulated a simple removal strategy in which hens were not harvested from either Buena Vista or Paul Olson unless the donor population contained at least 100 hens. However, even this limited removal of hens slightly increased the vulnerability of the donor populations (Figure 6). Moreover, in simulations where ≥1 population persisted in the CWGCA for 50 years, the average number of extant populations was slightly reduced unless translocations began relatively early, occurred frequently, and were used to reinforce Mead (Figure S4). Because investigating translocation effects on donor populations was not our principal objective, we did not systematically vary either the number of hens removed from donor populations or the requisite threshold to trigger a translocation event. However, conducting such analyses is straightforward and we encourage investigators, especially those working with small donor populations (e.g., many captive breeding programs), to further explore the effects of translocations on both donor and recipient populations in future studies.

Herein, we conducted the first population viability analysis for Greater Prairie‐Chickens in Wisconsin in the context of assessing the long‐term benefits of population reinforcement. Our results suggest that Buena Vista and Paul Olson are comparatively likely to persist for the next 50 years, whereas Leola and Mead are at high risk of quasi‐extirpation over the same time span even with the immediate introduction of 20 hens (more than doubling the estimated 2015 population size at each site). Although at least one population will almost certainly persist in the CWGCA for the next 50 years, a further range contraction is highly likely. However, we have also shown that an appropriate population reinforcement program can have a substantial effect on the long‐term viability of prairie‐chickens in the CWGCA, although the optimal strategy and the potential benefits of reinforcement can vary depending on which metric of population viability is targeted for management. We recognize that our method rests on a number of underlying assumptions, including constant carrying capacity, mean, and variance in population growth rate over time, uncorrelated environmental conditions, negligible demographic stochasticity, and minimal observation error in count data. Violating these assumptions can result in biased estimates of future extinction risk, for example, observation error can yield overinflated estimates of variation in population growth and overestimated extinction risk. Similarly, simulations that do not account for positive or negative autocorrelation in environmental conditions from one year to the next can lead to optimistic and pessimistic estimates of extinction risk, respectively. Nevertheless, count‐based PVA methods provide a useful tool for gauging the relative viability of two or more populations (Morris & Doak, 2002), or, as here, the relative viability of populations under a number of alternative management scenarios.

Our results demonstrate the value of clearly defining metrics of success, evaluating multiple alternative translocation strategies a priori to identify factors most strongly associated with success, and considering the long‐term effects of reinforcement on population persistence. The progress of reintroduction biology as a discipline will continue to hinge on case studies of specific translocation programs used for purposes of both reintroduction and reinforcement (Armstrong & Seddon, 2008), and future studies that incorporate a systematic model‐based approach such as the one described here or elsewhere in the literature (Armstrong & Reynolds, 2012; Converse & Armstrong, 2016) may eventually help elucidate broadly applicable relationships either within or between taxa. As most of the current literature deals largely with reintroductions, we strongly advocate future work focused specifically on population reinforcement, which may prove to be an essential tool for maintaining conservation‐dependent species.

## AUTHOR CONTRIBUTION

All authors worked together to conceptualize the project. M.A.H. conducted analyses and wrote the original draft of the manuscript with assistance from B.Z. S.D.H. secured funding and data for the project and provided logistical support. All authors contributed to writing and editing the final manuscript.
