# Supplementary material for: Swift action increases the success of population reinforcement for a declining prairie grouse
Source: Ecol Evol. 2018 Jan 15;8(3):1906–17. doi: 10.1002/ece3.3776 (PMC5792513; doi:10.1002/ece3.3776)
Supplement: Supplementary file 1 [file ECE3-8-1906-s001.docx]

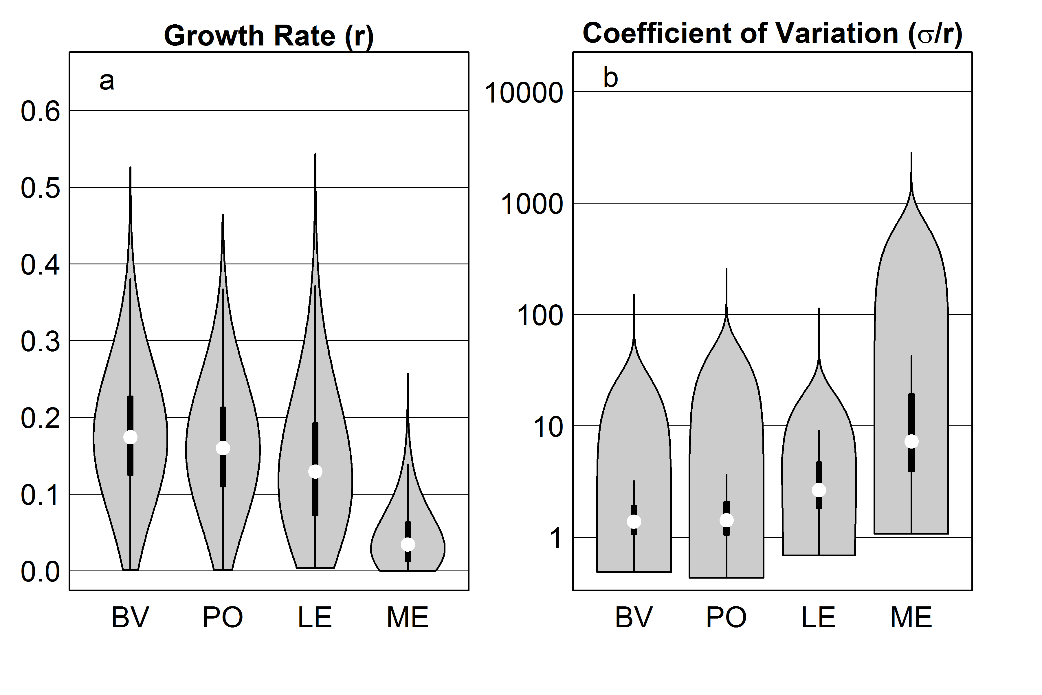


**Figure S1.** Intrinsic population growth rates (a) and variability in population growth (b) for four populations of Greater Prairie-Chickens in the Central Wisconsin Grassland Conservation Area, Wisconsin, USA (Buena Vista, BV; Paul Olson, PO; Leola, LE; Mead, ME). Populations with lower growth rates and larger coefficients of variation (e.g., LE, ME) are more vulnerable to quasi-extinction. Quantiles and distributions were estimated from 10,000 non-parametric bootstrap replicates.


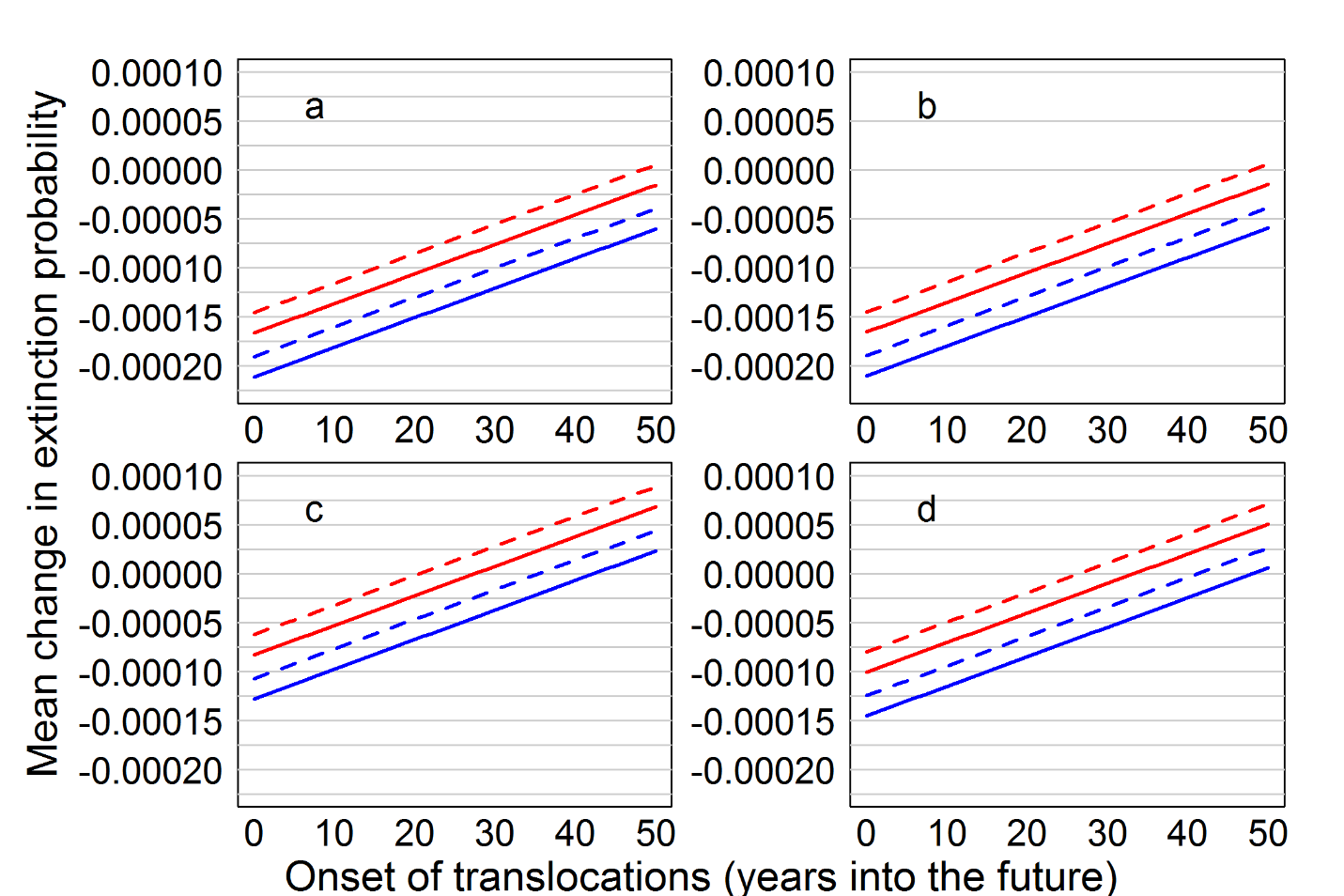


**Figure S2.** Simulated effects of translocations of Greater Prairie-Chickens originating from an outside source on regional quasi-extinction probability for four populations in the Central Wisconsin Grassland Conservation Area, Wisconsin, U.S.A. The best-supported model included onset of translocations, recipient population (Buena Vista, a; Paul Olson, b; Leola, c; Mead, d), frequency of translocations (decadal translocations, solid lines; single translocations, dashed lines), and level of translocation effort (100 individuals, blue; 20 individuals, red).


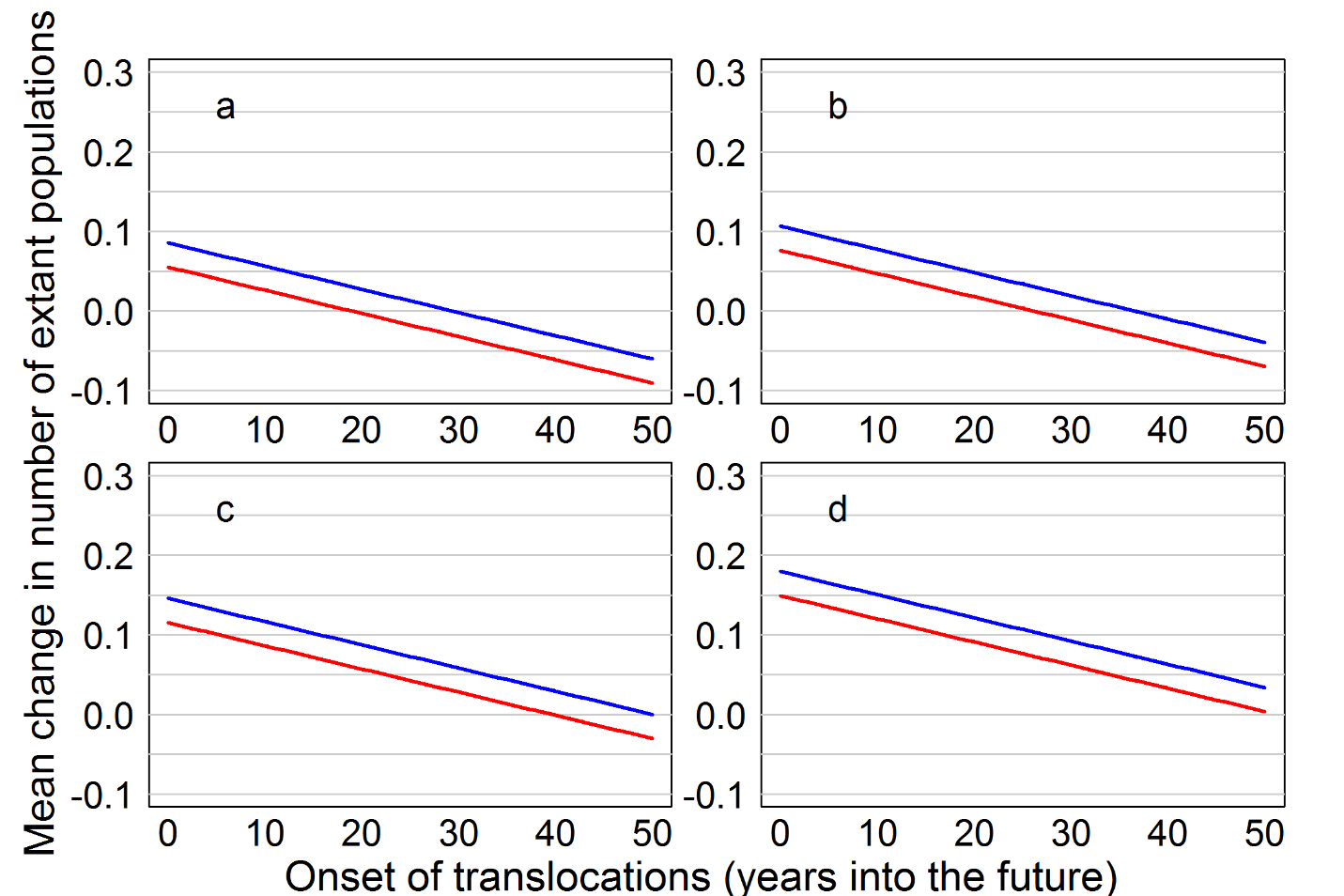


**Figure S3.** Simulated effects of translocations of Greater Prairie-Chickens originating from an outside source on the number of extant populations within the Central Wisconsin Grassland Conservation Area, Wisconsin, U.S.A. The best-supported model included onset of translocations, recipient population (Buena Vista, a; Paul Olson, b; Leola, c; Mead, d), and level of translocation effort (100 individuals, blue; 20 individuals, red).


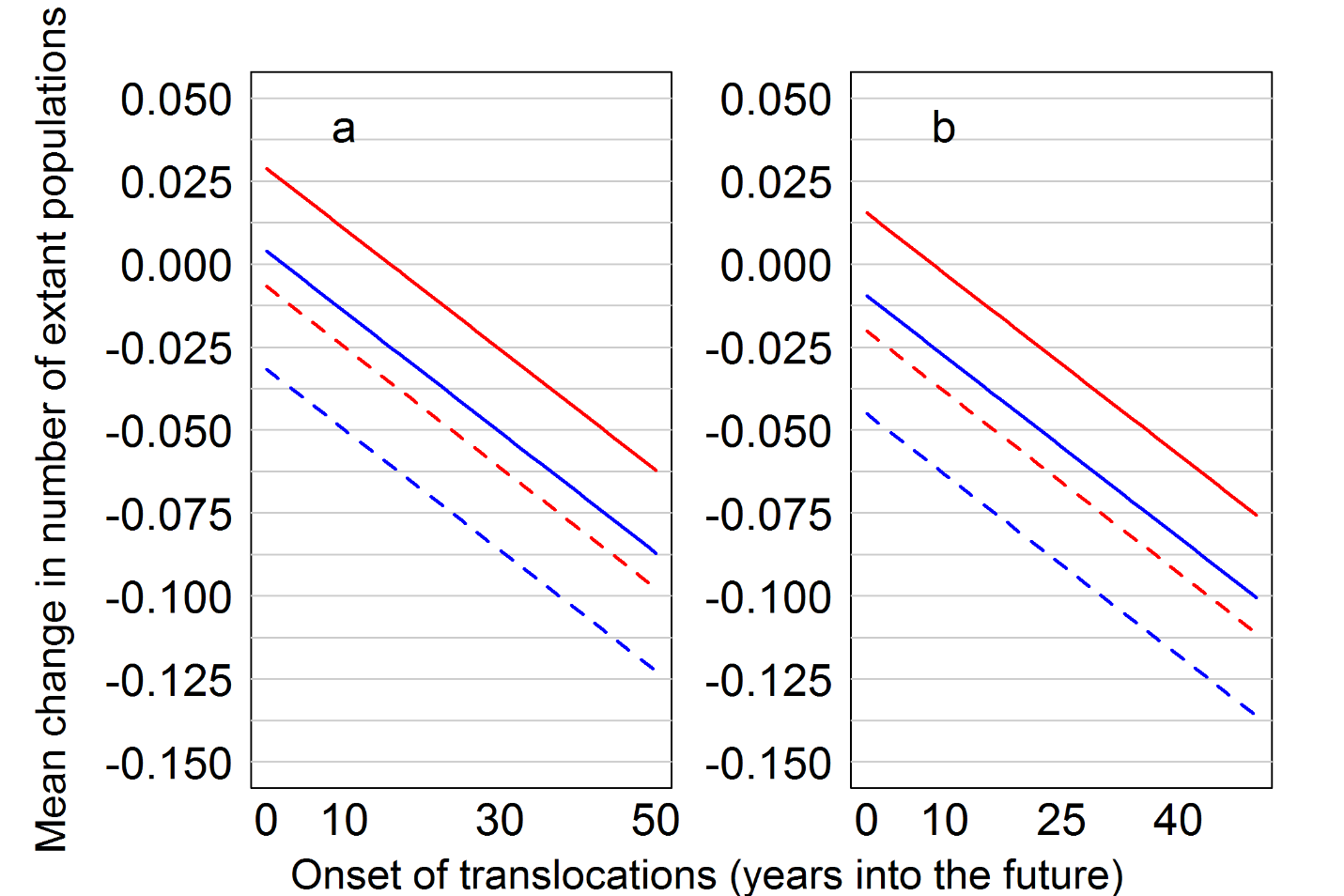


**Figure S4.** Simulated effects of translocations of Greater Prairie-Chickens from within the study area on the number of extant populations within the Central Wisconsin Grassland Conservation Area, Wisconsin, U.S.A. The best-supported model included onset of translocations, donor population (Buena Vista, a; Paul Olson, b), recipient population (Leola, blue; Mead, red), and frequency of translocation efforts (decadal translocations, solid lines; single translocations, dashed lines).
