# Supplementary material for: Swift action increases the success of population reinforcement for a declining prairie grouse
Source: Ecol Evol. 2018 Jan 15;8(3):1906–17. doi: 10.1002/ece3.3776 (PMC5792513; doi:10.1002/ece3.3776)
Supplement: Supplementary file 2 [file ECE3-8-1906-s002.docx]

**Table S1.** Number of male Greater Prairie-Chickens (*Tympanuchus cupido pinnatus*) counted by the Wisconsin Department of Natural Resources (WDNR) on leks at four core sites in central Wisconsin, U.S.A., 1950-2015. Prairie-Chicken survey reports are prepared annually by WDNR and are available to the public online at http://dnr.wi.gov.

| Year | Buena Vista | Leola | Paul Olson | Mead |
| --- | --- | --- | --- | --- |
| 1950 | 550 | 232 | - | - |
| 1951 | 550 | 183 | - | - |
| 1952 | 265 | 132 | - | - |
| 1953 | 344 | 146 | - | - |
| 1954 | 256 | 162 | - | - |
| 1955 | 305 | 110 | - | - |
| 1956 | 299 | 109 | - | - |
| 1957 | 239 | 114 | - | - |
| 1958 | 297 | 126 | - | - |
| 1959 | 169 | 72 | - | - |
| 1960 | 157 | 56 | - | - |
| 1961 | 135 | 46 | - | - |
| 1962 | 157 | 44 | 54 | - |
| 1963 | 150 | 37 | 50 | - |
| 1964 | 175 | 38 | 38 | - |
| 1965 | 165 | 21 | 43 | - |
| 1966 | 183 | 20 | 62 | - |
| 1967 | 141 | 10 | 66 | - |
| 1968 | 139 | 12 | 71 | - |
| 1969 | 104 | 28 | 57 | 43 |
| 1970 | 141 | 78 | 62 | 54 |
| 1971 | 198 | 77 | 47 | 102 |
| 1972 | 234 | 88 | 76 | 108 |
| 1973 | 155 | 46 | 94 | 121 |
| 1974 | 126 | 46 | 116 | 96 |
| 1975 | 138 | 52 | 135 | 118 |
| 1976 | 131 | 45 | 114 | 119 |
| 1977 | 213 | 75 | 145 | 154 |
| 1978 | 365 | 82 | 186 | 212 |
| 1979 | 438 | 53 | 189 | 211 |
| 1980 | 480 | 79 | 228 | 187 |
| 1981 | 550 | 75 | 302 | 180 |
| 1982 | 535 | 69 | 256 | 163 |
| 1983 | 359 | 49 | 188 | 97 |

**Table S1 (continued).** Number of male Greater Prairie-Chickens (*Tympanuchus cupido pinnatus*) counted by the Wisconsin Department of Natural Resources (WDNR) on leks at four core sites in central Wisconsin, U.S.A., 1950-2015. Prairie-Chicken survey reports are prepared annually by WDNR and are available to the public online at http://dnr.wi.gov.

| Year | Buena Vista | Leola | Paul Olson | Mead |
| --- | --- | --- | --- | --- |
| 1984 | 245 | 22 | 152 | 121^a^ |
| 1985 | 275 | 69 | 175 | 144 |
| 1986 | 194 | 47 | 152 | 127^a^ |
| 1987 | 193 | 56 | 194 | 110 |
| 1988 | 269 | 65 | 206 | 101 |
| 1989 | 182 | 64 | 124 | 128 |
| 1990 | 281 | 80 | 110 | 129 |
| 1991 | 216 | 84 | 91 | 101 |
| 1992 | 239 | 63 | 56 | 58 |
| 1993 | 265 | 65 | 93 | 65 |
| 1994 | 247 | 70 | 91 | 53 |
| 1995 | 275 | 87 | 83 | 38 |
| 1996 | 277 | 74 | 87 | 44 |
| 1997 | 334 | 97 | 100 | 59 |
| 1998 | 327 | 70 | 129 | 86 |
| 1999 | 341 | 89 | 139 | 92 |
| 2000 | 323 | 88 | 194 | 94 |
| 2001 | 252 | 69 | 174 | 69 |
| 2002 | 226 | 38 | 176 | 48 |
| 2003 | 269 | 34 | 183 | 49 |
| 2004 | 278 | 37 | 199 | 62 |
| 2005 | 229 | 22 | 129 | 51 |
| 2006 | 261 | 32 | 133 | 51 |
| 2007 | 327 | 38 | 181 | 53 |
| 2008 | 202 | 44 | 128 | 40 |
| 2009 | 178 | 42 | 109 | 26 |
| 2010 | 114 | 32 | 107 | 30 |
| 2011 | 136 | 31 | 111 | 19 |
| 2012 | 122 | 33 | 86 | 15 |
| 2013 | 126 | 37 | 119 | 13 |
| 2014 | 110 | 26 | 82 | 12 |
| 2015 | 133 | 17 | 90 | 13 |
| ^a^Surveys not conducted; values are the midpoint between the previous and following year's counts. | | | | |

**Table S2.** Model rankings, number of parameters (*K*), and Akaike weights (*w_i_*) for the effects of translocations on quasi-extinction probability for Greater Prairie-Chickens at four sites in the Central Wisconsin Grassland Conservation Area, Wisconsin, U.S.A. We considered level of effort (20 vs. 100 hens), frequency of translocations (single vs. decadal), and onset of translocations (5-45 years into the future).

**Table S3.** Model rankings, number of parameters (*K*), and Akaike weights (*w_i_*) for the effects of translocations on regional quasi-extinction probability for Greater Prairie-Chickens in the Central Wisconsin Grassland Conservation Area, Wisconsin, U.S.A. We considered level of effort (20 vs. 100 hens), frequency of translocations (single vs. decadal), onset of translocations (5-45 years into the future), and recipient population (BV, PO, LE, or ME).

**Table S4.** Model rankings, number of parameters (*K*), and Akaike weights (*w_i_*) for the effects of translocations on the number of extant populations of Greater Prairie-Chickens in the Central Wisconsin Grassland Conservation Area, Wisconsin, U.S.A. We considered level of effort (20 vs. 100 hens), frequency of translocations (single vs. decadal), onset of translocations (5-45 years into the future), and recipient population (BV, PO, LE, or ME).

**Table S5.** Model rankings, number of parameters (*K*), and Akaike weights (*w_i_*) for the effects of translocations on quasi-extinction probability for Greater Prairie-Chickens at four sites in the Central Wisconsin Grassland Conservation Area, Wisconsin, U.S.A. We considered frequency of translocations (single vs. decadal), onset of translocations (5-45 years into the future), recipient population (for Buena Vista and Paul Olson), and donor population (for Leola and Mead).

**Table S6.** Model rankings, number of parameters (*K*), and Akaike weights (*w_i_*) for the effects of translocations on regional quasi-extinction probability for Greater Prairie-Chickens in the Central Wisconsin Grassland Conservation Area, Wisconsin, U.S.A. We considered frequency of translocations (single vs. decadal), onset of translocations (5-45 years into the future), donor population (Buena Vista or Paul Olson), and recipient population (Leola or Mead).

**Table S7.** Model rankings, number of parameters (*K*), and Akaike weights (*w_i_*) for the effects of translocations on the number of extant populations of Greater Prairie-Chickens in the Central Wisconsin Grassland Conservation Area, Wisconsin, U.S.A. We considered frequency of translocations (single vs. decadal), onset of translocations (5-45 years into the future), donor population (Buena Vista or Paul Olson), and recipient population (Leola or Mead).
